# Reply: Evidence for heterogeneous nuclear ribonucleoprotein k over expression in oral squamous cell carcinoma

**DOI:** 10.1038/sj.bjc.6603912

**Published:** 2007-07-31

**Authors:** B Carpenter, G I Murray

**Affiliations:** 1 Auvation Ltd, Crombie Lodge, Aberdeen Science Park, Balgownie Drive, Aberdeen, UK; 2 Department of Pathology, University of Aberdeen, Aberdeen, UK

**Sir**,

Choudhury has shown, at the mRNA level using QPCR, that heterogeneous nuclear ribonucleoprotein k (hnRNP K) is overexpressed in oral squamous cell carcinoma. We identified hnRNP K as being upregulated in a different cancer, namely colorectal (Carpenter *et al*, 2006a), which strengthens the rationale that hnRNP K is important for tumorigenesis. Interestingly, using a tissue microarray with matching clinicopathological parameters, hnRNP K was described as being localised exclusively in the nucleus of normal colon cells whereas in colorectal tissues the protein was present in both the cytoplasmic and nuclear compartments (Carpenter *et al*, 2006b). The dysregulation of hnRNP K cellular localisation in colon cancer is suggestive that the protein has an altered cellular function, which is not surprising, as the protein has several cellular roles including, transcription, translation, mRNA shuttling and RNA editing.

As a result of matching clinicopathological data, our colon cancer study also revealed that hnRNP K protein expression increased as Dukes staging progressed and in Dukes C patients, high nuclear expression of hnRNP K was associated with good prognosis. As suggested by Choudhury, it will be interesting to determine if disease progression and prognosis correlates with hnRNP K protein expression in oral squamous carcinoma, as is the case for colorectal cancer. It will also be beneficial to expand these observations to different cancer types.

Overall, hnRNP K continues to emerge as an interesting protein, which through both mechanistic studies and expression profiling in different cancer types, has strongly been implicated as a key player of tumorigenesis. Validation through independent studies provides a strong portfolio of evidence that suggests hnRNP K is a good target for anticancer therapeutic and also holds the potential to be used as a diagnostic or prognostic marker.
